# L'Entente Cordiale

**Published:** 1904-10-08

**Authors:** 


### L'Entente Cordiale.

A number of French physicians and surgeons a few months ago expressed a desire to visit London in order to see the hospitals and laboratories and acquaint themselves with the organisation of the hospitals and the system of medical education in London. As science knows no nationality, and it is not often that the medical profession in England has an opportunity of welcoming its French confrères, a strong committee was at once formed, and arrangements were made to give our foreign guests a hearty reception. From small beginnings the party has increased very considerably. It was thought at first that perhaps as many as fifty Frenchmen might avail themselves of the opportunity of coming to England. It now appears that nearly 200 will land at Dover on Sunday evening and will stay in London at least till Thursday morning. They will be entertained in a variety of ways, and will be given the opportunity of seeing all they desire.

Physicians and surgeons in England are fairly well acquainted with the methods and teaching of the French, German, and Austrian schools, for Paris, Berlin, and Vienna lie within the compass of our travels, and we are in the habit of passing through these capitals in our journeys further afield. It is quite otherwise with the schools of the United Kingdom. There is no reason why a Frenchman or a German should put himself to the inconvenience of coming here. Big Congresses held at long intervals do indeed bring our continental brethren to England from time to time, but it would seem that the hurry and discomforts which are almost inseparable from such gatherings prevent the repetition of the visit. The present opportunity therefore is golden. A comparatively small number of men, all of the same nationality and many already known to each other makes the nucleus of a very agreeable and instructive party. We have too, much to show them in England both in object lessons, as to what to avoid and in examples which may seem worthy to be imitated. The wealth which is expended unostentatiously on our hospitals and the small amount granted by the State and municipal authorities may well excite their wonder, whilst the care lavished upon the patient as an individual and not as a mere case is the direct outcome of the independence which is so marked a trait in our national character.

Perhaps what will strike our friends first during their visits to the general hospitals will be the extreme care which is bestowed upon the training of nurses and the excellent manner in which they perform their duties. Next to this is the whole-hearted manner in which the students carry out their work both in the wards and the outpatient departments. The whole method of hospital work will be new and will require explanation. The system of voluntary support is unknown abroad, nor is there any feeling existent which would lead merchants, country gentlemen and others of similar position to give up two or three hours a week to the onerous and sometimes unpleasant duties of hospital management. The working of the Poor Law, too, so far as it concerns the sick pauper, must also be explained, and a visit for this purpose is arranged to one of the large infirmaries of whose existence and use even many Londoners are in ignorance. We think it highly probable that the keen observation which marks the work of French scientists may make them apprehend that owing to the great growth of late years in the number of surgical cases and the practice of our chief general hospitals to exclude nearly all but acute cases of disease, both students and nurses have not the necessary material in English clinical hospitals to complete their knowledge of disease and its treatment. This fact constitutes a grave defect in the facilities at present afforded to students and nurses by most of our great hospitals. If so a change for the better in the selection of cases for admission may result.

